# Extracellular Alkalinization as a Defense Response in Potato Cells

**DOI:** 10.3389/fpls.2017.00032

**Published:** 2017-01-24

**Authors:** Natalia Moroz, Karen R. Fritch, Matthew J. Marcec, Diwaker Tripathi, Andrei Smertenko, Kiwamu Tanaka

**Affiliations:** ^1^Department of Plant Pathology, Washington State University, PullmanWA, USA; ^2^Agricultural and Food Systems, Washington State University, PullmanWA, USA; ^3^Molecular Plant Sciences Program, Washington State University, PullmanWA, USA; ^4^Institute of Biological Chemistry, Washington State University, PullmanWA, USA

**Keywords:** extracellular alkalinization, apoplastic pH, potato, suspension cell culture, defense response

## Abstract

A quantitative and robust bioassay to assess plant defense response is important for studies of disease resistance and also for the early identification of disease during pre- or non-symptomatic phases. An increase in extracellular pH is known to be an early defense response in plants. In this study, we demonstrate extracellular alkalinization as a defense response in potatoes. Using potato suspension cell cultures, we observed an alkalinization response against various pathogen- and plant-derived elicitors in a dose- and time-dependent manner. We also assessed the defense response against a variety of potato pathogens, such as protists (*Phytophthora infestans* and *Spongospora subterranea*) and fungi (*Verticillium dahliae* and *Colletotrichum coccodes*). Our results show that extracellular pH increases within 30 min in proportion to the number of pathogen spores added. Consistently with the alkalinization effect, the higher transcription level of several defense-related genes and production of reactive oxygen species was observed. Our results demonstrate that the alkalinization response is an effective marker to study early stages of defense response in potatoes.

## Introduction

The innate immunity of plants allows them to resist a wide variety of biotic stresses. Plants sense potential pathogen attacks by recognizing conserved molecules among microbes, so-called pathogen-associated molecular patterns (PAMPs), while plants are also able to sense damaged-self by recognizing damage-associated molecular pattern (DAMPs) which are released from plant cells upon damage or pathogen infection. PAMPs and DAMPs induce plant defenses, pattern-triggered immunity, which is qualitatively similar to those activated during the gene-for-gene resistance or effector-triggered immunity (Jones and Dangl, 2006).

Pattern-triggered immunity is a set of multiple early defense responses directed to increase plant resistance against stress. Plant stress responses vary significantly across different hosts and pathogens (Atkinson and Urwin, 2012; Rejeb et al., 2014). These differences include the production of plant hormones, reactive oxygen species (ROS), and nitric oxide, as well as dynamic changes in ion balance leading to increases in cytosolic calcium levels and rapid extracellular alkalinization (Felle, 2001; Nurnberger and Scheel, 2001; Torres et al., 2006; Bellin et al., 2013; Seybold et al., 2014; Wu et al., 2014; Verma et al., 2016). Furthermore, transcriptional changes in stress-responsive genes lead to the synthesis of pathogenesis-related proteins and the production of low molecular mass secondary metabolites, e.g., phytoalexins (Ahuja et al., 2012) and antimicrobial peptides (Brotman et al., 2009).

The potato is one of the world’s most important vegetable crops. Many diseases caused by various biotic and abiotic factors significantly affect the yield and quality of the potatoes produced (Kapsa, 2008). Although recognition of potato tuber diseases during pre- or non-symptomatic phases is important, little is known about pathogen-induced defense responses in potatoes. Only a few methods have been reported for evaluation of potato defense responses; e.g., ROS assays and expression of pathogen-related genes (Niebel et al., 1995; Kolomiets et al., 2000; Arseneault et al., 2004; Kobayashi et al., 2007; Halim et al., 2009; Sánchez et al., 2010; Gao et al., 2013; Wiesel et al., 2015). In addition, measurement of phytoalexins such as rishitin, lubimin, and solavetivone (Tomiyama et al., 1968; Brindle et al., 1983) is a direct way to evaluate plant defense response. However, these assays are time consuming and require specialized equipment or reagents which may not be available to many labs.

Analysis of stress responses in plant organs can be confounded by tissue-specific responses. To overcome this potential caveat, suspension cell culture systems are commonly used as a simple and highly reproducible system to study plant stress responses. For example, soybean cell cultures were used to detect intracellular calcium changes in response to fungal spores (Navazio et al., 2007), while tobacco suspension cells were used to analyze calcium-based signaling induced by pathogen- and plant-derived elicitors (Manzoor et al., 2012). Moreover, pine, tobacco, *Arabidopsis* and grapevine suspension cells were used to study plant and microbial peptides involved in innate immune responses (Popp et al., 1997; Pearce et al., 2001, 2008; Fammartino et al., 2007; Shirron and Yaron, 2011; Chang and Nick, 2012). Tomato suspension cells were used to study fungal virulence factors (Masachis et al., 2016), the replication of potato spindle tuber viroids (Zelcer et al., 1981), and the effect of general elicitors on the accumulation of phosphatidic acid (van der Luit et al., 2000). Finally, sweet potato suspension cells were used to study defense signaling peptides (Chen et al., 2008) and early stress responses by the yeast elicitor invertase (Debarry et al., 2005). There are a few limitations in the use of the suspension culture system, which requires maintenance of aseptic condition and occasionally does not reflect the *in vivo* situation, i.e., heterogeneous. However, given its simplicity and high reproducibility of data, the suspension culture is the most convenient system for obtaining accurate results.

Here, we propose a method to measure early response to stress in potato suspension culture cells using extracellular alkalinization as proxy. The rationale for this approach is that extracellular alkalinization is one of the earliest responses to biotic stress (Wu et al., 2014). The objective of our work here is to create a simple, reliable, and fast method to detect a defense response in potato cells. Our results suggest that the alkalinization assay is a powerful method to evaluate early plant defense response against pathogen-derived and plant-derived elicitors as well as external pathogens.

## Materials and Methods

### Elicitors

Potato, tomato and pepper systemins (Constabel et al., 1998) were a generous gift from Gregory Pearce. Flg22 (22 amino acids flagellin peptide), Elf26 (conserved amino terminus of bacteria elongation factor EF-Tu) and AtPEP (plant elicitor peptides from *Arabidopsis*) were synthesized by GenScript, Inc. (Piscataway, NJ, USA). Chitin 6-mer and OGA (oligogalacturonic acid; degree of polymerization = 10–15) were synthesized by Elicityl-OligoTech (France). Chitin mixture (from shrimp shells) and ATP were obtained from Sigma-Aldrich.

### Pathogen Spore Preparation and Quantification

*Phytophthora infestans* was grown on rye agar (Ribeiro, 1978) for 7 days at 23°C with a 16 h light cycle. Fresh leaves of potato (cv. Russet Burbank) were rinsed with deionized water and placed on a glass tray lined with a moist paper towel and mesh to minimize possible cross-contamination. To initiate *P. infestans* sporulation, rye agar plugs (5-mm in diameter) were placed on the adaxial side of the potato leaves. The tray was covered with a transparent plastic bag, sealed and incubated at 15°C for 7–10 days in the dark. Potato leaves with late blight (black/brown) lesions were rinsed with sterile deionized water. To release the zoospores sporangia suspension was incubated for 2 h in the dark at 4°C.

*Spongospora subterranea* inoculum was prepared from potato root galls collected by Dr. Dennis Johnson from a commercial potato field in Washington State in 2001. The inoculum was prepared using a modification of a previously reported method (Merz, 1989). To obtain cystosori of *S. subterranea*, the powder of the infected root gall tissue was resuspended in Hoagland’s solution No 2, pH 7.5 (Caisson Laboratories, Inc., Smithfield, UT, USA) and incubated for 6 days at room temperature with orbital shaking at 150 rpm in the dark.

The fungi, *Verticillium dahliae* and *Colletotrichum coccodes*, were grown in media with a half-strength of Potato Dextrose Agar (BD Difco) for 10–15 days (Melouk, 1992; Carnegiea et al., 2003). Grown mycelium was scraped from the plate surface and resuspended in 3–5 mL of sterile deionized water. The suspension was filtered through a layer of Kimwipes.

Pathogens spores/spore balls were quantified using a hemocytometer (Hausser Scientific, Horsham, PA, USA) under a light microscope (ICC50 HD, Leica) at 40–60x magnification.

### Maintenance of Suspension Cells Cultures

Potato suspension cell culture derived from potato tuber (*Solanum tuberosum* L. cv. Russet Burbank) was kindly provided by Dr. Jeffrey Suttle (Law and Suttle, 2005). The cell suspension was grown in Murashige and Skoog (MS) medium (pH 5.8) containing 4.3 g/L MS salt with Vitamins (Caisson Laboratories, Inc.), 30 g/L (w/v) sucrose, 0.5 mg/L (w/v) α-Naphthaleneacetic acid (NAA), and 1 mg/L (w/v) 2,4-dichlorophenoxyacetic acid (2,4-D). The potato cells were grown at 23°C with orbital shaking at 130 rpm in the dark. The potato cell suspension was maintained in 250 ml flasks by transferring 30 mL of cell culture to 60 mL of fresh medium every 7 days.

Potato suspension cells images were done using light microscope (DMI 3000 B, Leica). The size of the potato cells at days 3 and 7 after passaging was calculated as mean ± SE of 100 cells using ImageJ software^1^.

*Arabidopsis* T87 cells, obtained from *Arabidopsis* Biological Resource Center (Columbus, OH, USA), were cultured aseptically in NT-1 medium containing 4.3 g/L (w/v) MS salt, 30 g/L (w/v) sucrose, 0.18 g/L (w/v) KH_2_PO_4_, 1 mg/L (w/v) thiamine, 5 mg/L (w/v) 2,4-D, and 100 mg/L (w/v) myo-Inositol (pH 5.8 adjusted with 5 N NaOH). The cell culture was grown at room temperature (22–26°C) with orbital shaking at 130 rpm in the light. The cell suspension was maintained by transferring 4 mL of the cell culture to 76 mL of fresh medium every 7 days.

### Measurement of Apoplastic pH

Seven-day old suspension cell culture was transferred into fresh medium. Three to 4 days after transfer, 1.3 mL of suspension potato cells were aliquoted into each well of 24-well cell culture plates (Greiner Bio-One, max volume 3.3 mL). The cells experience a spike in pH after they are aliquoted, and they equilibrate to pH 4.5–5.0 after 2–4 h of shaking at room temperature, 180 rpm. Elicitors and pathogen spores were resuspended in sterile deionized water to make stock solutions. The pH of the pathogens spores’ suspensions was adjusted to that in the equilibrated potato cells culture (pH 4.5–5.0). The stock solutions of elicitors and pathogen spores were then diluted stepwise with sterile deionized water to obtain the desired concentrations. The equal volumes of all solutions of elicitors or pathogen spores (or water only as control treatment) were applied to the suspension cells. The added volumes varied from 10 to 600 μL depending on the elicitor/pathogen spores stock concentrations. The pH was recorded using an Accumet AB15 basic pH-meter with an accuTupH electrode (Fisher Scientific). Changes in the extracellular pH (ΔpH) were calculated using the following equation:

$$\begin{array}{c} \Delta pH=({\mathrm{pH}}_{\mathrm{sample}}-{\mathrm{pH}}_{\mathrm{control}})\;\pm \;\;\delta \\ \;\delta =\;\sqrt{{\left(\delta_{\mathrm{sample}}\right)}^{2}+{\left(\delta_{\mathrm{control}}\right)}^{2}}\; \end{array}$$

where pH_control_ and pH_sample_ are an average pH and δ is the standard error (mean ± SE of three replicates in three independent experiments). The pH of potato cell suspensions after application of only sterile deionized water was used as a control. For the time-dependent experiments, the change in pH was compared to the control at each time point respectively. To exclude a possible false positive pH shift, pathogen spore suspensions were tested using MS medium only (without potato cells); alkalinization of MS medium in the presence of the same concentrations of pathogens was not observed. Student’s *t*-test was performed to determine a significant difference between samples.

### Luminol-Based Oxidative Burst Assay

Potato suspension culture cells (200 μL) were transferred to a single well of a white 96-well microplate (PerkinElmer). For the assay 10x solution consists of 1 mM of L-012, highly sensitive luminol derivative (Wako Chemicals USA, Inc.) and 200 μg/mL of horseradish peroxidase (Sigma-Aldrich) with or without elicitors (10 mM chitin or 0.25 mM systemin) or pathogens spores (1 × 10^5^ spores/mL of *V. dahliae, C. coccodes*, and *P. infestans;* or 1 × 10^5^ spore balls/mL of *S. subterranea*) was used. Mock treatment (as a control) was performed by applying the equal volume of sterile deionized water. After 2 h of cells’ pre-incubation at room temperature (no shaking), 22 μL 10x assay solution, was added and luminescence from each well was measured during 1 s for each time point using an EnSpire multimode plate reader (PerkinElmer).

### Quantitative Real-Time Reverse Transcription (qRT)-PCR

After treatment of potato culture cells by elicitors or pathogens, the supernatant was discarded and the cells were frozen in liquid nitrogen and stored at -80°C. Total RNA was extracted using TRIzol reagent (Invitrogen). The frozen potato cells were partially thawed on ice and then crushed with ceramic beads for 30 s using Mini-Beadbeater (Biospec). Debris was pelleted by centrifugation for 10 min at 13,000 rpm, 4°C (Centrifuge 5415R, Eppendorf). Chloroform was mixed with the supernatant (1/5 volume of TRIzol used) and the suspension centrifuged for 10 min at 13,000 rpm, 4°C. Total RNA was precipitated from the supernatant by adding 100% isopropanol (1/2 volume of TRIzol used). The RNA pellet was washed with 70% Ethanol (same volume of TRIzol used) followed by centrifugation. Total RNA was resuspended in 100 μL of Milli-Q RNase free water, and its concentration quantified. First strand cDNA was synthesized from 1 μg total RNA using an iScript cDNA Synthesis Kit (Bio-Rad). Real time RT-PCR was performed using SsoAdvanced Universal SYBR Green Supermix Kit (Bio-Rad) with a CFX96 Real-Time System (Bio-Rad). Sequence information of the forward and reverse primers for the reference genes and the potato defense-related genes are listed in **Table 1**. Cytoplasmic ribosomal protein L2 (*L2*) and ubiquitin (*Ubq*) were used as reference genes for expression data normalization. Cq (quantification cycle) was estimated from a linear regression fit through the points of the log-linear phase of the amplification curve. Using the Cq value, gene expression levels relative to a reference gene were calculated for each sample using the following equation (Schmittgen and Livak, 2008):

**Table 1:** Primers sequences for potato housekeeping genes (L2 and Ubq) and defense genes used in this study.

Gene	Accession no.	5′–3′ sequence	Reference
L2	39816659	F: GGCGAAATGGGTCGTGTTAT	Nicot et al., 2005
		R: CATTTCTCTCGCCGAAATCG	
Ubq	BQ045862	F: CTCCGTGGTGGTATGCAGAT	Gonzalez-Lamothe et al., 2008
		R: CACGTTGTCAATGGTGTCG	
PAL-1	X63103	F: TTGCACAAGTTGCATCCATT	Wang et al., 2008
		R: CACCAGCTCTTGCACTTTCA	
PAL-2	X63104	F: GGTCACTGCCTCGGGTGAT	Arseneault et al., 2004
		R: CCTGCCAGTGAGCAAACCA	
PR-1b	AY050221	F: GGCATCCCGAGCACAAAAT	Arseneault et al., 2004
		R: CTGCACCGGAATGAATCAAGT	
PR-5	AY737317.1	F: GGAGGCAGACGACTCGACTT	Arseneault et al., 2004
		R: CCATGGTTGTTCCTGGATTCA	
HMG-2	AB041031	F: ACAAGAAGCCAGCAGCAGTT	Wang et al., 2008
		R: CCACAAGAGCAGCAACTTCA	
WRKY	DMG402007388	F: AAAATATGGTCAAAAAGTGACAAGAG	Wiesel et al., 2015
		R: CATGTTGGTGCAAATGAACAC	

$$2^{-\Delta Cq}=\frac{2^{-\Delta Cq^{\mathrm{Sample}}}}{2^{-\Delta Cq^{\mathrm{Ref}}}}.$$

Statistical analysis of the values of three biological replicates was performed by Student’s *t*-test to calculate probability of induction or repression. The mean value of the control treatment was used to determine the fold change in transcript level.

## Results

### Optimization of Alkalinization Assay Conditions Using Potato Suspension Cells

**FIGURE 1:**
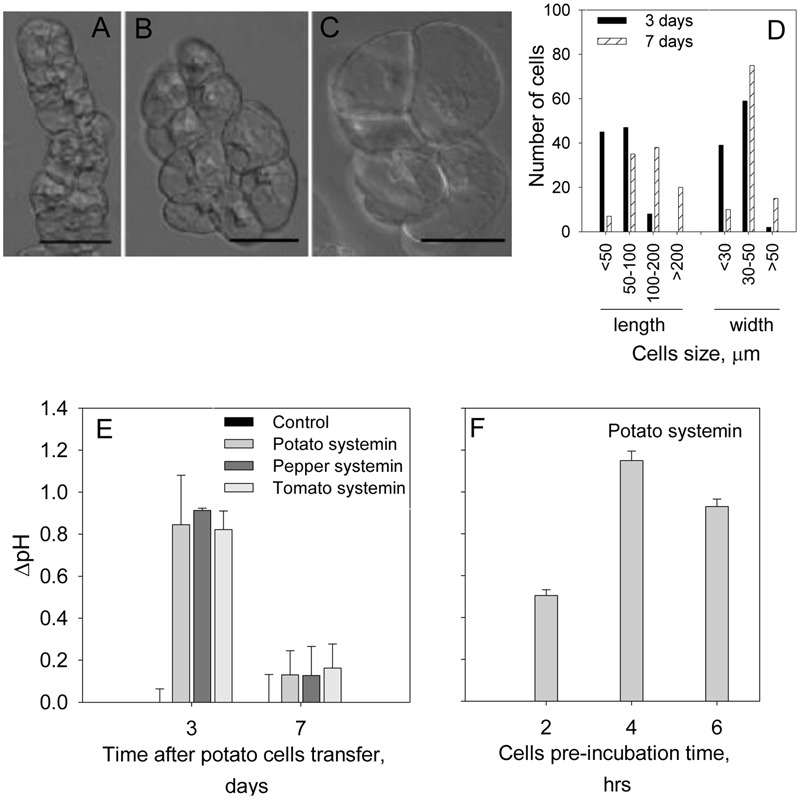
**Optimization of conditions for potato suspension cells to use for the alkalinization assay.** Photographs show representative light microscope images of the potato suspension cells: **(A)** at day 3; **(B,C)** at day 7 after transfer for subculture. Scale bars = 50 μm. Note that the morphology was not changed during 7 days after passaging the cells while cells size was increased. **(D)** The length and width of 100 cells at each time point after passaging was measured under the microscope. **(E)** Effect of the days after passaging the cells on the efficiency of the alkalinization assay in the presence of 2 μM of potato, pepper and tomato systemins. **(F)** Efficiency of the alkalinization assay as a function of the cells’ pre-incubation time prior to the treatment with 2 μM potato systemin. Histograms show mean ± SE of three replicates in three independent experiments.

We first checked the range of pH of the MS medium (5.5–6.0) to get optimal efficiency of the alkalinization assay, and found that pH 5.8 was the optimal for both cell growth and the sensitivity of the alkalinization assay. To evaluate the optimal dilution factor for the cells passaging, the cells culture derived from potato tuber was diluted every 7 days with MS fresh medium at the ratios culture:medium 1:10 or 1:3. The highest pH response was detected at 1:3 dilution. The time after cells transfer was also evaluated. In these assays we treated cells culture after 3 or 7 days of the passaging with a known potato DAMP, systemin. Although, time after cell transfer did not change potato cells morphology (**Figures 1A–C**), the cell size was increased and swollen cells were observed (**Figures 1B,C**). As shown in **Figure 1D**, majority of cells at day 7 were >100 μm in length, whereas <100 μm at day 3. The width of the potato cells was 1.2 times greater in average (40.29 ± 10.85 μm at day 7 in comparison to 33.52 ± 7.34 μm at day 3), while the length was 2.3 times longer in average (137.74 ± 75.28 μm at day 7 in comparison to 60.06 ± 27.75 μm at day 3). The alkalinization response of the potato cells against systemin was markedly higher in cells at day 3 after passaging (**Figure 1E**). We further evaluated the pre-incubation time at room temperature before recording the pH. We carried out the experiments after 2, 4, and 6 h of pre-incubation and found that the most efficient extracellular alkalinization can be detected after 4 h of pre-incubation in the culture plate (**Figure 1F**) and adhered to this time in the following assays.

### Time- and Dose-Dependent Effects of Various Elicitors on the Extracellular pH

**FIGURE 2:**
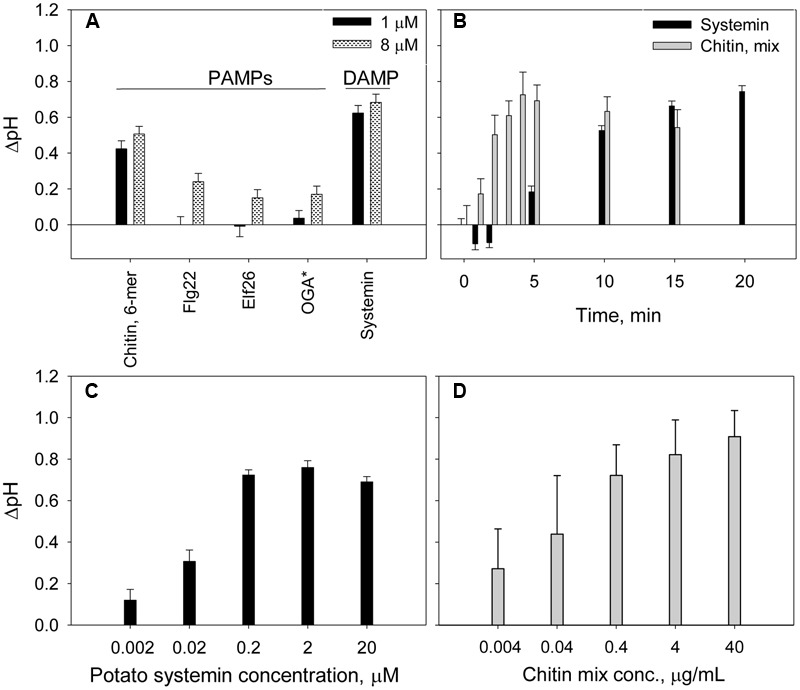
**Time- and dose-dependent extracellular alkalinization of potato suspension cells in the presence of elicitors. (A)** Effect of PAMP and DAMP elicitors on the extracellular pH changes. ^∗^OGA concentrations were 1 μg/mL (black) and 10 μg/mL (gray pattern). **(B)** Effect of 2 μM of potato systemin and 4 μg/mL of chitin oligomers mixture on extracellular pH over 20 min. **(C,D)** Alkalinization effect of potato systemin and chitin mixture, respectively, at various concentrations. The pH was recorded after 15 and 5 min of suspension cells incubation with systemin and chitin, respectively. Histograms show mean ± SE of three replicates in three independent experiments.

Elicitors are molecules known to trigger plant defense in plants (Hahn, 1996) and are generally classified as pathogen-derived elicitors, PAMPs, and plant-derived elicitors, DAMPs. We tested several PAMPs (purified chitin 6-mer, Flg22, and Elf26) and DAMP (systemin and OGA) at two different concentrations. Systemin and chitin produced a significantly higher alkalinization effects when compared to the other elicitors (**Figure 2A**). Although, Flg22 and Elf26 is widely used in plant defense studies (Felix et al., 1999; Kunze et al., 2004), no alkalinization was observed for potato suspension cells when these elicitors were added at 1 μM concentration. The scarce, but significant increase of extracellular pH was detected in response to 8 μM of Flg22, Elf26 (**Table 2**). OGA was reported to induce extracellular alkalinization at concentrations 7.5–50 μg/mL (Spiro et al., 2002). However, for the potato suspension cells, the alkalinization effect by OGA addition was small (ΔpH was 0.12 and 0.17 at the concentration of 10 and 50 μg/mL, respectively). Further, we performed the time-dependent experiment with chitin and systemin as representatives of the elicitor groups since their strong alkalinization effects. Results showed that extracellular alkalinization was initiated in the first few minutes after addition of chitin mixture, and reach their maximum within 5 min followed by a slow reduction during next 15 min (**Figure 2B**). In contrast, alkalinization is slowly culminated in approximately 15–20 min in the presence of systemin. This kinetic difference was observed at the other concentrations we tested. To demonstrate the sensitivity of our alkalinization assay, we measured effect of systemin and chitin in a dose-dependent response. A marked alkalinization was observed in cultures exposed to even 2 nM of systemin or 4 ng/mL of chitin mixture (**Figures 2C,D**).

**Table 2:** Comparison of the alkalinization effect of various elicitors on the potato and *Arabidopsis* suspension cells.

Elicitor		Elicitor concentration	ΔpH^§^	
			Potato suspension cells	*Arabidopsis* suspension cells
PAMPs	Flg22	1 μM	0.05 ± 0.028	0.29 ± 0.029^∗∗∗^
		8 μM	0.08 ± 0.039^∗^	0.53 ± 0.031^∗∗∗^
	Elf26	1 μM	0.08 ± 0.050	0.33 ± 0.240^∗∗∗^
		8 μM	0.15 ± 0.046^∗^	0.64 ± 0.033^∗∗∗^
	Chitin 6-mer	8 μM	0.51 ± 0.042^∗∗∗^	0.71 ± 0.283^∗∗∗^
DAMPs	OGA	10 μg/mL	0.12 ± 0.045^∗∗^	0.17 ± 0.039^∗^
	ATP	500 μM	0.21 ± 0.048^∗^	0.41 ± 0.032^∗∗∗^
	AtPep1	1 μM	0.08 ± 0.048	0.76 ± 0.256^∗∗∗^
	Potato systemin	0.25 μM	0.84 ± 0.044^∗∗∗^	0.12 ± 0.047^∗^

*^§^The alkalinization was recorded in 5 min after ATP addition, in 60 min after OGA addition and in 15 min for all other elicitors.*

*^∗^*P* < 0.05; ^∗∗^0.001 < *P* < 0.01; ^∗∗∗^*P* < 0.001 compared to the corresponding values of each control treatment.*

Next we examined host-specific alkalinization effects caused by identical elicitors. We exposed potato and *Arabidopsis* suspension cells to PAMPs (Flg22, Elf26, and chitin mixture) and DAMPs (OGA, ATP, AtPEP, and systemin). As shown in **Table 2**, the alkalinization effect of AtPEP from *Arabidopsis* is significantly stronger in *Arabidopsis* suspension cells, while systemin from potato stimulated alkalinization only in potato suspension cells. These results are consistent with previous reports showing species specificity in the peptidic DAMPs (Lori et al., 2015).

### Dynamic Changes in Extracellular pH in Response to Potato Pathogens

**FIGURE 3:**
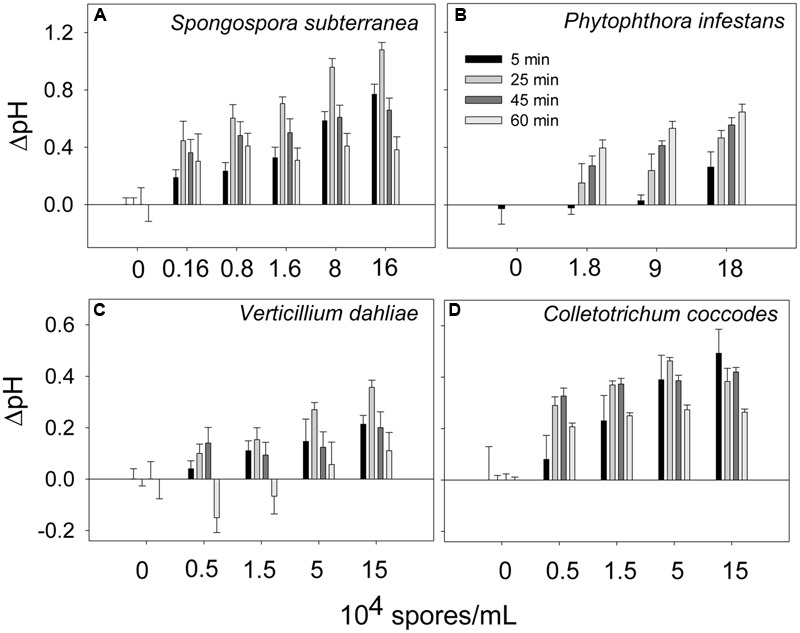
**Time- and dose-dependent extracellular alkalinization of potato suspension cells in the presence of potato pathogens.** The pH was recorded in 5, 25, 45, and 60 min after addition of spores of *Spongospora subterranea* **(A)**, *Phytophthora infestans* **(B)**, *Verticillium dahliae* **(C)**, and *Colletotrichum coccodes* **(D)**. Histograms show mean ± SE of three replicates in three independent experiments.

Extracellular alkalinization was monitored in the presence of several potato pathogens: *S. subterranea, P. infestans, V. dahliae*, and *C. coccodes*. The pathogens were added to the potato suspension cells at the indicated concentrations and the pH was recorded. As shown in **Figure 3**, the levels of extracellular alkalinization increased in a dose-dependent manner. The bell-shaped time-dependent alkalinization response peaked at 25 min (ΔpH = 0.35–1.0) when the potato cells were incubated in the presence of all pathogens (**Figures 3A,C,D**), except for *P. infestans*. In the presence of *P. infestans* the extracellular pH was gradually increased in during 60 min of cells incubation at all tested pathogen concentrations (**Figure 3B**).

### Oxidative Burst Response in Potato Suspension Cells

**FIGURE 4:**
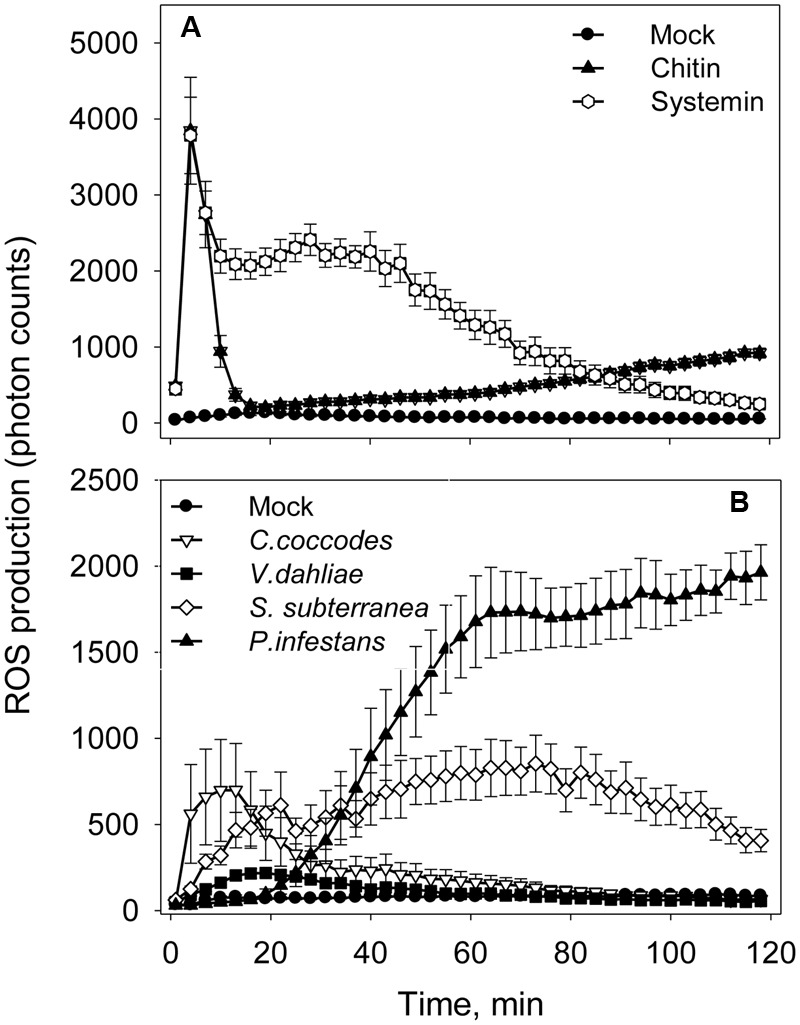
**Elicitors- and pathogens-induced reactive oxygen species (ROS) accumulation in potato cells. (A)** Time-dependent ROS production in the presence of elicitors (10 μM of Flg22, Elf26, or chitin 6-mer; 0.5 mM of ATP or 1 μM Systemin). **(B)** Time-dependent ROS production in the presence of pathogens (1 × 10^5^ spores/mL of *V. dahliae, C. coccodes*, or *P. infestans*; or 1 × 10^5^ spore balls/mL of *S. subterranea*). Data show photon counts in 1.0 s at each time point with mean ± SE (*n* = 6).

An oxidative burst is the rapid release of ROS from stressed plant cells upon contact with pathogens. This robust method is sensitive enough to capture dynamic changes in ROS production at an early time point in infected tissues (Smith and Heese, 2014). To detect this early ROS response, a luminol-based assay was performed in the presence of the same elicitors and pathogens as in alkalinization assay (**Figure 4**). In consistence with the alkalinization assay, ROS production was not observed when Flg22 (1 and 10 μM), Elf26 (1 and 10 μM), and ATP (0.5 mM) were added to potato cell suspension (Supplemental Figure S1). Chitin 6-mer and fungal pathogens (*V. dahliae* and *C. coccodes*) induced oxidative bursts within first 10 min (**Figures 4A,B**). Systemin and *S. subterranea* induced broader peaks of ROS production in comparison to those induced by the other treatments (**Figures 4A,B**). Interestingly, in the presence of *P. infestans* the increase in ROS level was observed only after 20 min of incubation (**Figure 4B**).

### Expression of Defense-Related Genes in Potato Suspension Cells

**FIGURE 5:**
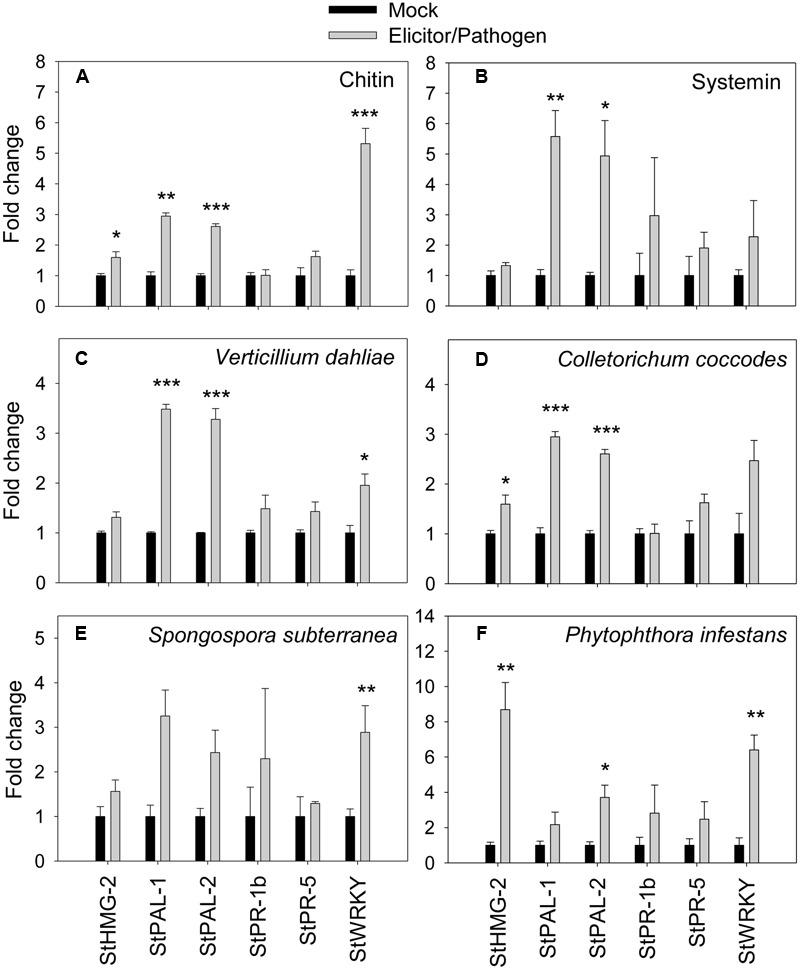
**Effects of elicitors and pathogens on expression of defense-related genes.** Amplification of potato defense-related genes were monitored by qRT-PCR after elicitation of potato suspension cells with chitin 6-mer **(A)**, potato systemin **(B)**, *V. dahliae* **(C)**, *C. coccodes* **(D)**, *S. subterranea* **(E)**, and *P. infestans* **(F)**. Histograms show the normalized expression data with mean ± SE of three biological replications. ^∗^*P* < 0.05; ^∗∗^0.001 < *P* < 0.01; ^∗∗∗^*P* < 0.001 compared to the corresponding values of each control treatment.

To confirm the correlation between the observed extracellular alkalinization and pathogen response, we quantified transcription of defense-related genes by qRT-PCR in total RNA from potato cells treated with elicitors or pathogens for 60 min (**Figure 5**). The results show that expressions of salicylic acid-responsive *PAL-1, PAL2*, and *WRKY* (similar to *AtWRKY40*; Wiesel et al., 2015) were upregulated by 2.5 times or more in the presence of systemin and chitin relatively to the non-treated control. Transcription of *HMG-2* and *PR-5* was only slightly upregulated by both elicitors (**Figures 5A,B**). Expression of *PR-1b* was not affected by chitin, however, was increased by three times in the presence of systemin. Effect of both fungal pathogens, *V. dahliae* and *C. coccodes*, was very similar; expression of *PAL-1, PAL-2*, and *WRKY* was increased by twofold and more, however the upregulation of *HMG-2, PR-1b*, and *PR-5* was not prominent (**Figures 5C,D**). *S. subterranea* led to significant upregulation of *PAL-1, PAL2*, and *WRKY*, while the expression of *PR-1b, PR-5*, and *HMG-2* genes was changed slightly (**Figure 5E**). In the contrast with other pathogens, transcription of *HMG-2* was highly upregulated (eight times) in the presence of *P. infestans*. Expression of *WRKY, PR-1b, PR-5*, and *PAL-1* was significantly increased, while no changes were observed in *PAL-2* expression (**Figure 5F**).

## Discussion

A combination of different detection methods for the whole spectrum of plant defense responses will facilitate unraveling complexity of the molecular processes underlying immunity in potatoes. Several methods have been applied for evaluating defense responses in potatoes including measurements of ROS production, secondary metabolite accumulations (oxylipins, phytoalexins, etc.), and transcriptional expression of defense-related genes (Niebel et al., 1995; Kolomiets et al., 2000; Arseneault et al., 2004; Kobayashi et al., 2007; Halim et al., 2009; Sánchez et al., 2010; Sapko et al., 2011; Gao et al., 2013; Wiesel et al., 2015). In the present study, we propose a method for measuring extracellular alkalinization in response to elicitors and pathogens using potato suspension cell culture. Changes in the extracellular alkalinization is one of the earliest responses to biotic stresses (Felix et al., 1999) and could be used for the early identification of disease during pre- or non-symptomatic phases. Although, alkalinization assays have been successfully used to identify and characterize plant-derived elicitors in a number of species (Pearce and Ryan, 2003; Scheer et al., 2003; Huffaker et al., 2006; Pearce et al., 2009, 2010; Chang and Nick, 2012), their usefulness in potato system has not been evaluated thus far. Here, we demonstrate applicability of extracellular alkalinization assay for the potato suspension cell culture.

Assays using suspension cell cultures are a useful system to study plant defense responses. Several applications using potato suspension cells have been reported for studies of plant stress responses. For example, leaf-derived potato suspension cells were used to study resistance to osmotic stress (Sabbah and Tal, 1990) and to measure accumulation of phytoalexins (Brindle et al., 1983), biosynthesis of oxylipins (Stumpe et al., 2001), nitric oxide and ROS production (Sapko et al., 2011), and expression of defense-related genes (Monjil et al., 2014). Most notably, exposure of suspension cells established from potato leaf protoplasts to cutin monomers induces alkalinization, production of ethylene, and transcriptional up-regulation of the defense-related genes (Schweizer et al., 1996). In our work, the alkalinization was detected in response to both PAMPs and DAMPs and also to spores of pathogens. This point out versatility of the potato suspension culture cells for evaluating different types of the response. In addition, our study expands the capability of alkalinization assays by showing reliable fast responses against pathogens and damage.

Another important aspect of our study is optimization conditions for an efficient alkalinization assay using potato suspension cells. It has been shown that characteristics of the suspension cell cultures could depend on the type of initiating tissues (Law and Suttle, 2005; Lecourieux et al., 2005; Navazio et al., 2007; Pearce et al., 2008; Manzoor et al., 2012). Furthermore, efficiency of an assay depends significantly on the age of the culture (Popp et al., 1997). As shown in **Figure 1**, the most efficient conditions for the maximum extracellular alkalinization response were observed 3 d after the passaging and with a lower dilution.

We demonstrated the high sensitivity of the alkalinization assay using potato cell culture in the presence of elicitors, such as chitin and systemin, which correlates with the published data on the extracellular alkalinization of *Arabidopsis* suspension cells after treatment with synthetic AtPep1 (Pearce et al., 2010), tomato cells with RALF, Rapid Alkalinization Factor (Masachis et al., 2016), and tobacco cells with synthetic ultrashort cationic lipopeptides (Brotman et al., 2009) at nanomolar elicitors’ concentrations. Relatively higher concentrations of the well-known bacterial elicitors Flg22 and Elf26 (in comparison to the other elicitors we tested) were required to induce alkalinization in potato cell suspensions when compared with that for *Arabidopsis* (**Figure 2**; **Table 2**). One possible explanation is that our cell culture was derived from tubers which express lower levels of the corresponding receptors than above-ground organs. Plausibly, compatible combination of the elicitor and a host species ought to be used to achieve the strongest response. Tissue-specific and species-selective elicitation has been already reported for several elicitors, for example: (1) Flg22 from different sources induces distinct defense response in tobacco, tomato, and potato plants (Hao et al., 2014); (2) in *Arabidopsis*, shoots and roots respond to chitin and Pep1, whereas roots are insensitive to Elf26 and only had a minor increase in Ca^2+^ levels in response to Flg22 (Ranf et al., 2011); (3) another group of elicitors, fungal glucans, triggers defense responses in various plants, including tobacco, rice, tomato and potato with different efficiency (Fesel and Zuccaro, 2016); (4) the level of defense genes expression for potato foliage and tuber is different under the treatment of the late blight pathogen *P. infestans* (Gao and Bradeen, 2016). These results suggest that the mechanism of PAMP recognition depends on the plant tissues and species, and further studies of defense responses using potato cells derived from different tissues can help to elucidate these various mechanisms.

Although, extracellular alkalinization is an essential defense response, the nature of the response remains unknown. It has been attributed to a modification of plasma membrane permeability for calcium ions, protons, potassium ions, and anion fluxes that can follow changes in extracellular pH (Felix et al., 1999). Alternatively, alkalinization can be caused by secretion of cationic protein: a theory supported by a recent report showing that a rust-induced secreted protein in the poplar tree is a small cationic antifungal protein that induces extracellular alkalinization (Petre et al., 2016). Identification of a protein or ion channel responsible for extracellular alkalinization in potato will further accelerate our understanding of molecular mechanisms of plant defense and eventually contribute to engineering disease resistance into potatoes and other crops.

## Author Contributions

NM designed the experiments, performed the experiments, analyzed and interpreted the data, wrote and edited the paper. MM, DT, KF, and AS performed the experiments, reviewed the manuscript and wrote comments. KT conceived and designed the experiments, analyzed and interpreted the data, revised the work critically for important intellectual content, approved the final version of the paper to be published.

## Conflict of Interest Statement

The authors declare that the research was conducted in the absence of any commercial or financial relationships that could be construed as a potential conflict of interest.

## Footnotes

1 https://imagej.nih.gov/ij/
