# Supplementary material for: Extracellular Alkalinization as a Defense Response in Potato Cells
Source: Front Plant Sci. 2017 Jan 24;8:32. doi: 10.3389/fpls.2017.00032 (PMC5258701; doi:10.3389/fpls.2017.00032)
Supplement: Supplementary file 1 [file Data_Sheet_1.pdf]

Supplemental Figure S1

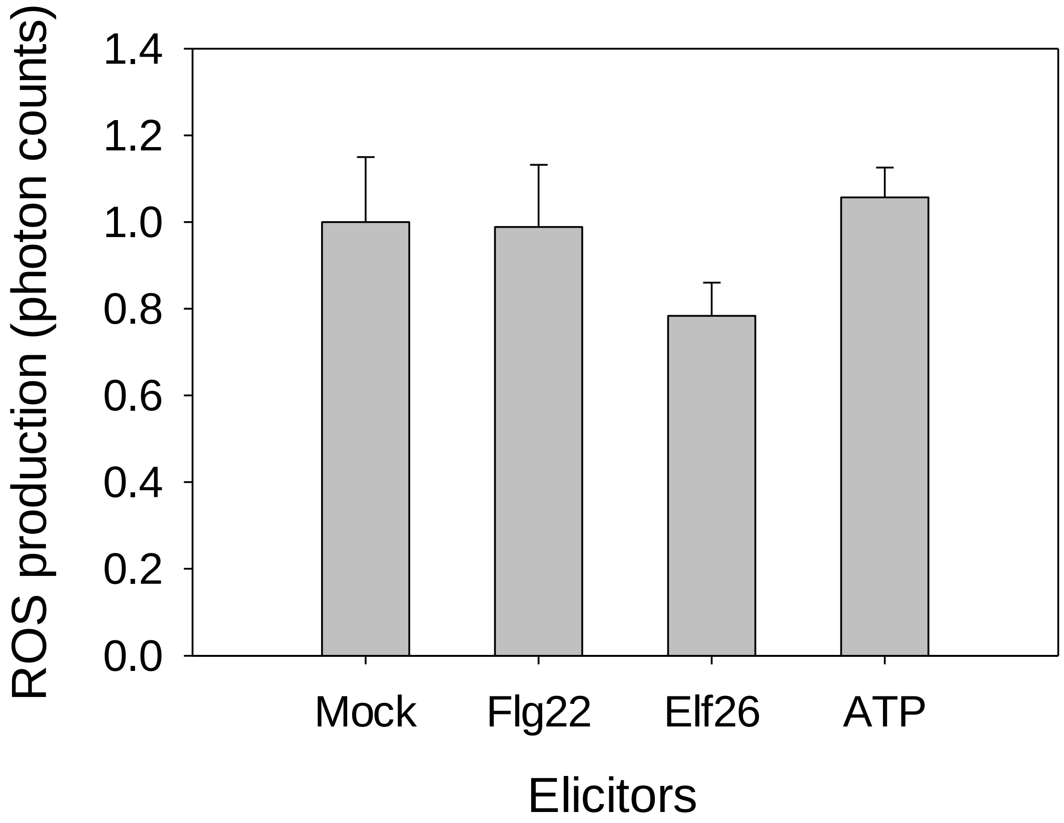

Figure S1. ROS accumulation was not induced in the presence of the elicitors Flg22, Elf26, and ATP. Histogram shows the normalized data with mean ± SE (n = 6) for total ROS production in 120 min upon addition of the elicitors 10 µM of Flg22 or Elf26, or 0.5 mM of ATP in the potato cells.
